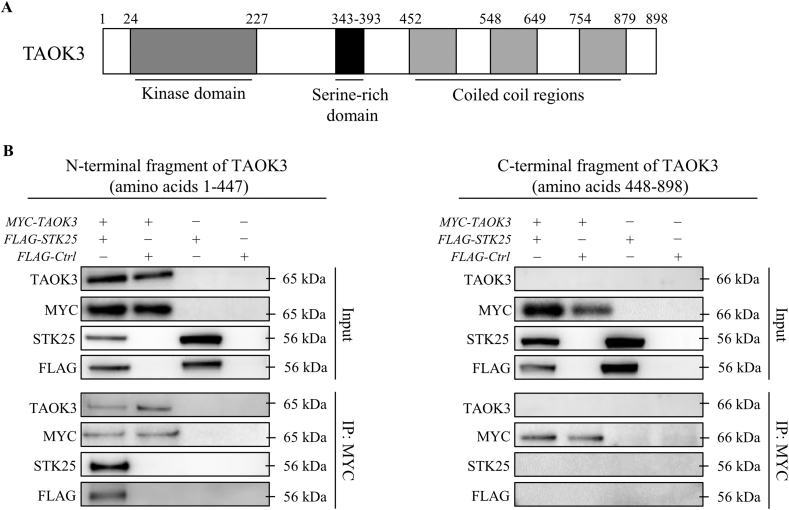# STE20-type kinase TAOK3 regulates hepatic lipid partitioning

**DOI:** 10.1016/j.molmet.2021.101353

**Published:** 2021-10-08

**Authors:** Ying Xia, Mara Caputo, Emmelie Cansby, Sumit Kumar Anand, Silva Sütt, Marcus Henricsson, Rando Porosk, Hanns-Ulrich Marschall, Matthias Blüher, Margit Mahlapuu

**Affiliations:** 1 Department of Chemistry and Molecular Biology, University of Gothenburg and Sahlgrenska University Hospital, Gothenburg, Sweden; 2 Biomarker Discovery and Development, Research and Early Development, Cardiovascular, Renal, and Metabolism (CVRM), BioPharmaceuticals R&D, AstraZeneca, Gothenburg, Sweden; 3 Department of Molecular and Clinical Medicine/Wallenberg Laboratory, Institute of Medicine, University of Gothenburg and Sahlgrenska University Hospital, Gothenburg, Sweden; 4 Department of Biochemistry, Institute of Biomedicine and Translational Medicine, University of Tartu, Tartu, Estonia; 5 Department of Medicine, University of Leipzig, Leipzig, Germany

**Keywords:** TAOK3, Nonalcoholic fatty liver disease, Nonalcoholic steatohepatitis, Liver lipid metabolism, Intrahepatocellular lipid droplets

## Abstract

**Objective:**

Nonalcoholic fatty liver disease (NAFLD), defined by excessive lipid storage in hepatocytes, has recently emerged as a leading global cause of chronic liver disease. The aim of this study was to examine the role of STE20-type protein kinase TAOK3, which has previously been shown to associate with hepatic lipid droplets, in the initiation and aggravation of human NAFLD.

**Methods:**

The correlation between *TAOK3* mRNA expression and the severity of NAFLD was investigated in liver biopsies from 62 individuals. In immortalized human hepatocytes, intracellular fat deposition, lipid metabolism, and oxidative and endoplasmic reticulum stress were analyzed when TAOK3 was overexpressed or knocked down by small interfering RNA. Subcellular localization of TAOK3 was characterized in human and mouse hepatocytes by immunofluorescence microscopy.

**Results:**

We found that the *TAOK3* transcript levels in human liver biopsies were positively correlated with the key lesions of NAFLD (i.e., hepatic steatosis, inflammation, and ballooning). Overexpression of TAOK3 in cultured human hepatocytes exacerbated lipid storage by inhibiting β-oxidation and triacylglycerol secretion while enhancing lipid synthesis. Conversely, silencing of TAOK3 attenuated lipid deposition in human hepatocytes by stimulating mitochondrial fatty acid oxidation and triacylglycerol efflux while suppressing lipogenesis. We also found aggravated or decreased oxidative/endoplasmic reticulum stress in human hepatocytes with increased or reduced TAOK3 levels, respectively. The subcellular localization of TAOK3 in human and mouse hepatocytes was confined to intracellular lipid droplets.

**Conclusions:**

This study provides the first evidence that hepatic lipid droplet-coating kinase TAOK3 is a critical regulatory node controlling liver lipotoxicity and susceptibility to NAFLD.

## Highlights

• *TAOK3* levels in human liver biopsies correlate positively with the severity of NAFLD.
• The subcellular localization of TAOK3 in human and mouse hepatocytes is confined to intracellular lipid droplets.
• The overexpression or silencing of TAOK3 in human hepatocytes results in exacerbated or reduced, respectively, lipid accumulation as well as oxidative and endoplasmic reticulum stress.

## Abbreviations

^1^H-MRS: single-proton magnetic resonance spectroscopy
4-HNE: 4-hydroxynonenal
8-oxoG: 8-oxoguanine
ADRP: adipose differentiation-related protein
CCl_4_: carbon tetrachloride
CDAA: choline-deficient l-amino-acid
CHOP: C/EBP-homologous protein
DEN: diethylnitrosamine
DHE: dihydroethidium
ER: endoplasmic reticulum
IHHs: immortalized human hepatocytes
JNK: c-Jun-N terminal kinase
NAFLD: nonalcoholic fatty liver disease
NAS: NAFLD activity score
NASH: nonalcoholic steatohepatitis
NTC: non-targeting control
PBS: predicted biological score
PEX5: peroxisomal biogenesis factor 5
PMP70: peroxisomal membrane protein 70 kDa
si: small interfering
T2D: type 2 diabetes
TAG: triacylglycerol
TAOK3: thousand and one kinase 3
VLDL: very low-density lipoprotein
Y2H: yeast two-hybrid
YAP: Yes-Associated Protein

## 1 Introduction

Nonalcoholic fatty liver disease (NAFLD) is defined as lipid accumulation in >5% of hepatocytes (steatosis) in the absence of excessive alcohol use (≥30 g per day for men and ≥20 g per day for women) [1]. Recently, NAFLD has emerged as a leading global cause of chronic liver disease due to the exponential rise in obesity, which is the main risk factor for the development and aggravation of NAFLD [2]. Importantly, about 10–20% of patients with NAFLD progress to nonalcoholic steatohepatitis (NASH), which is characterized by local inflammation and cellular injury (ballooning) in the liver, in addition to fat infiltration [3]. Patients with NASH are at high risk of developing cirrhosis, liver failure, and hepatocellular carcinoma (HCC), which is one of the most fatal and fastest-growing cancers [4]. Thus, understanding the molecular pathogenesis of NAFLD is of high clinical importance for the efficient prevention and management of a range of complex liver diseases.

In NAFLD, lipids accumulate within intrahepatocellular lipid droplets composed of a neutral core of triacylglycerol (TAG) and cholesterol esters, surrounded by a phospholipid monolayer that harbors a unique set of proteins [5]. Liver lipid droplet-associated proteins are increasingly recognized as critical regulators of not only hepatic lipid metabolism but also protein quality control and storage, cell signaling, viral replication, and interactions with other organelles [6]. Notably, among genetic variants that confer susceptibility to NAFLD, the best characterized are a single-nucleotide polymorphism in the *PNPLA3* gene and a splice variant (rs72613567:TA) in the *HSD17B13* gene, both of which encode proteins anchored to the liver lipid droplets [7,8]. Furthermore, we have recently demonstrated that several STE20-type kinases – STK25, MST3, and MST4 – associate with intrahepatocellular lipid droplets and critically orchestrate liver lipid partitioning and NAFLD development [[9], [10], [11], [12], [13], [14], [15], [16], [17]]. Consequently, mapping the composition of the lipid droplet proteome in hepatocytes and exploring its mode of action in the control of liver lipid homeostasis are essential for deciphering the molecular pathophysiology of NAFLD.

Our recent studies using global proteomic analysis have identified thousand and one kinase 3 (TAOK3; also known as MAP3K18, JIK, or DPK) as a lipid droplet-associated protein in mouse liver [14,15]. TAOK3 is a STE20-type kinase, widely expressed in different cell types and previously linked to the regulation of MAPK signaling [18,19]. The role of TAOK3 in the immune system is emerging as it has recently been reported to regulate the commitment to the marginal zone B cell fate [20], terminal differentiation of conventional dendritic cells [21], and canonical TCR signaling [22]. The role of TAOK3 has also been implicated in tumor initiation and metastasis formation in pancreatic cancer and reduced cell death in breast cancer [18,23]. However, the function or mode of action of this kinase in hepatocytes has not been investigated yet.

In this study, we provide several lines of evidence suggesting a possible role of TAOK3 in the initiation and aggravation of human NAFLD. We found that *TAOK3* mRNA levels in human liver biopsies correlate positively with the severity of NAFLD. Furthermore, we show that the overexpression or silencing of TAOK3 in human hepatocytes results in exacerbated or reduced, respectively, lipid accumulation as well as oxidative and endoplasmic reticulum (ER) stress. In line with our previous observations in mouse liver [14,15], we found that the subcellular localization of TAOK3 is confined to intracellular lipid droplets in human hepatocytes.

## 2 Materials and methods

### 2.1 Analysis of the *TAOK3* mRNA expression in human liver biopsies

The *TAOK3* mRNA expression was analyzed in interoperative liver biopsies collected from Caucasian subjects (men, *n* = 35; women, *n* = 27) who underwent laparoscopic abdominal surgery for Roux-en-Y bypass (*n* = 12), sleeve gastrectomy (*n* = 9), or elective cholecystectomy (*n* = 41). The participants fulfilled the following inclusion criteria: (1) men and women aged >18 years; (2) indication for elective laparoscopic or open abdominal surgery; (3) BMI between 18 and 50 kg/m^2^; (4) abdominal MRI feasible; and (5) signed written informed consent. The exclusion criteria for liver biopsy donors were as follows: (1) significant acute or chronic inflammatory disease or clinical signs of infection; (2) CrP >10 mg/dl; (3) type 1 diabetes and/or antibodies against glutamic acid decarboxylase (GAD) and islet cell antibodies (ICA); (4) systolic blood pressure >140 mmHg and diastolic blood pressure >95 mmHg; (5) clinical evidence of cardiovascular or peripheral artery disease; (6) thyroid dysfunction; (7) alcohol or drug abuse; and (8) pregnancy. Type 2 diabetes (T2D) was diagnosed by a fasting plasma glucose value >7.0 mmol/l and/or a 120-min oral glucose tolerance test (OGTT) glucose value >11.1 mmol/l. For participant characteristics, refer to Cansby et al. [15].

Body fat was analyzed by dual X-ray absorptiometry (DEXA) and liver fat was measured by single-proton magnetic resonance spectroscopy (^1^H-MRS) as described previously [24]. A small liver biopsy was obtained during the surgery (between 08:00 and 10:00 h after overnight fasting), immediately snap frozen in liquid nitrogen, and stored at −80 °C. The NAFLD activity score (NAS) and fibrosis score were assessed on liver sections by a certified pathologist [25]. qRT-PCR was performed in liver biopsies as described below using the probes for TAOK3 (Hs00937694_m1) and 18S rRNA (Hs99999901_s1; Thermo Fisher Scientific, Waltham, MA), which span exon–exon boundaries to improve the specificity.

All subjects gave written informed consent to use their data in anonymized form for research purposes before taking part in this study. All investigations were approved by the Ethics Committee of the University of Leipzig, Germany (approval numbers 363-10-13,122,010 and 159-12-21,052,012), and were performed in accordance with the Declaration of Helsinki.

### 2.2 Cell culture

Immortalized human hepatocytes (IHHs; a gift from B. Staels, the Pasteur Institute of Lille, University of Lille Nord de France, Lille, France) [26] were maintained in Complete William's E Medium (GlutaMAX supplemented; Gibco, Paisley, UK), supplemented with 10% (vol/vol) FBS, 1% (vol/vol) penicillin/streptomycin (Gibco), human insulin (20 U/l; Actarapid Penfill; Novo Nordisk, Bagsværd, Denmark), and dexamethasone (50 nmol/l; Sigma–Aldrich, St. Louis, MO). The cells were demonstrated to be free of mycoplasma infection by using the MycoAlert Mycoplasma Detection Kit (Lonza, Basel, Switzerland).

### 2.3 Transient overexpression and RNA interference

IHHs were transfected with the human *MYC*-tagged *TAOK3* expression plasmid (EX-A8822-M43; GeneCopoeia, Labomics, Nivelles, Belgium) or an empty control plasmid (EX-NEG-M43; GeneCopoeia) using Lipofectamine 2000 (Thermo Fisher Scientific). Truncated versions of the *MYC*-tagged *TAOK3* expression plasmid were generated using the Q5 Site-Directed Mutagenesis Kit (New England Biolabs, Ipswich, MA) and verified by sequencing. The cells were also transfected with *TAOK3* small interfering (si)RNA (Hs.644,420; Ambion, Austin, TX) or scrambled siRNA (SIC001; Sigma–Aldrich) using Lipofectamine RNAiMax (Thermo Fisher Scientific). 24 h after the transfections, the culture medium was replaced by fresh medium, with or without supplementation with 50 μmol/l oleic acid (Sigma–Aldrich), for subsequent 48-hour incubation.

### 2.4 Assessment of lipid metabolism, mitochondrial function, and oxidative stress

IHHs were stained with Bodipy 493/503 (Invitrogen, Carlsbad, CA), MitoTracker Red and Green (Thermo Fisher Scientific), or dihydroethidium (DHE; Life Technologies, Grand Island, NY) as described previously [14]. In parallel, IHHs were processed for immunofluorescence with anti-TAOK3, anti-adipose differentiation-related protein (ADRP), anti-8-oxoguanine (8-oxoG), anti-4-hydroxynonenal (4-HNE), anti-E06, anti-KDEL, anti-C/EBP-homologous protein (CHOP), anti-peroxisomal biogenesis factor 5 (PEX5), or anti-peroxisomal membrane protein 70 kDa (PMP70) antibodies (see Supplementary Table S1 for antibody information). Immunofluorescence images were acquired using a Zeiss Axio Observer microscope with the ZEN Blue software (Zeiss, Oberkochen, Germany). The labeled area was quantified in 6 randomly selected microscopic fields (×20) per well using the ImageJ software (1.47v; National Institutes of Health, Bethesda, MD).

Membrane lipids were extracted using the BUME method [27]; free cholesterol was quantified using straight-phase HPLC with an evaporative light scattering detector (ELD) as previously described [28], while sphingomyelin, phosphatidylcholine, lysophosphatidylcholine, and phosphatidylethanolamine were measured using direct infusion on a QTRAP 5500 mass spectrometer (Sciex, Concord, Canada) equipped with a robotic nanoflow ion source (TriVersa NanoMate; Advion BioSciences, Ithaca, NJ) [29].

To measure β-oxidation, IHHs were incubated in the presence of (9,10-^3^H[N])palmitic acid (PerkinElmer, Waltham, MA), and [^3^H]-labeled water was quantified as the product of free fatty acid oxidation [11]. TAG secretion and incorporation of [^14^C]glucose (PerkinElmer) into TAGs were assessed as described previously [9]. Fatty acid uptake was measured using the Quencher-Based Technology (QBT) Fatty Acid Uptake Assay Kit (Molecular Devices, San Jose, CA). Cell viability was detected using the CellTiter-Blue Cell Viability Assay (Promega, Stockholm, Sweden) according to the manufacturer's recommendations. The TAG hydrolase activity was determined in total cell lysates using [^3^H]triolein (PerkinElmer) as the substrate [17].

Targeted metabolomics was carried out to analyze phosphohexoses and amino acids by multiple reaction monitoring scan on a QTRAP 4500 mass spectrometer (Sciex, Concord, Canada) as described previously [30].

### 2.5 qRT-PCR, Co-Immunoprecipitation, and Western blot

RNA was isolated from tissue samples and IHHs with the RNeasy Lipid Tissue Mini Kit (Qiagen, Hilden, Germany) or the EZNA Total RNA Kit (Omega Bio-Tek, Norcross, GA). cDNA was synthesized using the High-Capacity cDNA Reverse Transcription Kit (Thermo Fisher Scientific). Relative quantification was performed with the QuantStudio 6 Flex Real-Time PCR System (Thermo Fisher Scientific) or the CFX Connect Real-Time System (Bio-Rad, Hercules, CA). The relative quantities of the target transcripts were calculated after normalization of the data to the endogenous control, 18S rRNA (Thermo Fisher Scientific). Co-immunoprecipitation was carried out using anti-MYC antibodies according to the manufacturer's instructions (Anti-c-MYC Magnetic Beads; Thermo Fisher Scientific). Western blot analysis was performed as described previously [31] (see Supplementary Table S1 for antibody information).

### 2.6 Yeast two-hybrid analysis

Yeast two-hybrid (Y2H) screening was performed by Hybrigenics Services, S.A.S., Evry, France (http://www.hybrigenics-services.com) as described below. The coding sequence for *Homo sapiens* STK25 (NM_006374.5; positions 181 to 1461) was PCR-amplified and cloned into pB27 as a C-terminal fusion to the LexA DNA-binding domain (LexA-STK25) and into pB35 as a C-terminal fusion to the Gal4 DNA-binding domain (Gal4-STK25). The constructs were checked by sequencing and used as a bait to screen a random-primed primary human hepatocyte cDNA library constructed into pP6. pB27 and pP6 were derived from the original pBTM116 [32] and Pgadgh [33] plasmids, respectively. pB35 was constructed by inserting the Gal4 DNA-binding domain from pAS2ΔΔ [34] into the pFL39 backbone [35] under the control of the MET25 promoter [36].

For the LexA-STK25 bait construct, 66 million clones (5.5-fold the complexity of the library) were screened using a mating approach with YHGX13 (Y187 ade2-101::loxP-kanMX-loxP, matα) and L40ΔGal4 (mata) yeast strains as described previously [34]. 35 His+ colonies were selected on a medium lacking tryptophan, leucine, and histidine. For the Gal4-STK25 bait construct, a total of 74 million clones (6.1-fold the complexity of the library) were screened using the same mating approach with YHGX13 (matα) and CG1945 (mata) yeast strains. 81 His+ colonies were selected on a medium lacking tryptophan, leucine, methionine, and histidine. The prey fragments of the positive clones were amplified by PCR and sequenced at their 5′ and 3’ junctions. The resulting sequences were used to identify the corresponding interacting proteins in the GenBank database (NCBI) using a fully automated procedure.

A confidence score (PBS, for predicted biological score) was attributed to each interaction as described previously [37]. Briefly, the PBS relies on two different levels of analysis. First, a local score takes into account the redundancy and independency of the prey fragments, as well as the distribution of reading frames and stop codons in the overlapping fragments. Second, a global score takes into account the interactions found in all the screens performed at Hybrigenics using the same library. This global score represents the probability of an interaction being nonspecific. For practical use, the PBS scores were divided into four categories, from A (highest confidence) to D (lowest confidence). A fifth category (E) specifically flags interactions involving highly connected prey domains previously found several times in screens performed on libraries derived from the same organism. Finally, several of these highly connected domains have been confirmed as false-positives of the technique and are tagged as F. The PBS scores have been shown to positively correlate with the biological significance of the interactions [38,39].

### 2.7 Statistical analysis

The statistical significance between the groups was evaluated using the two-sample Student's *t*-test with a value of *P* < 0.05 considered statistically significant. The correlation between the *TAOK3* expression in human liver biopsies and the hepatic lipid content, NAS, and fibrosis score was investigated by Spearman's rank correlation analysis after the assessment of normality of data using the Kolmogorov–Smirnov test. All statistical analyses were conducted using SPSS statistics (v27; IBM Corporation, Armonk, NY).

## 3 Results

### 3.1 *TAOK3* expression in the human liver is positively correlated with the severity of NAFLD

NAFLD is defined by the excessive accumulation of fat in the liver [1]. Thus, we first analyzed the correlation between the expression of *TAOK3* mRNA in liver biopsies and the hepatic fat content measured by magnetic resonance spectroscopy (^1^H-MRS) in a cohort of 62 participants representing a wide range of BMI (22.7–45.6 kg/m^2^) and body and liver fat content (19.5–57.9% and 1.1–50.0%, respectively). We found that the hepatic *TAOK3* mRNA abundance was positively correlated with the liver fat levels (Figure 1A). Notably, there was no correlation between the *TAOK3* transcript and the gender, BMI, body fat, waist-to-hip ratio, or HbA1c values of the subjects (Supplementary Figure S1).

**Figure 1:**
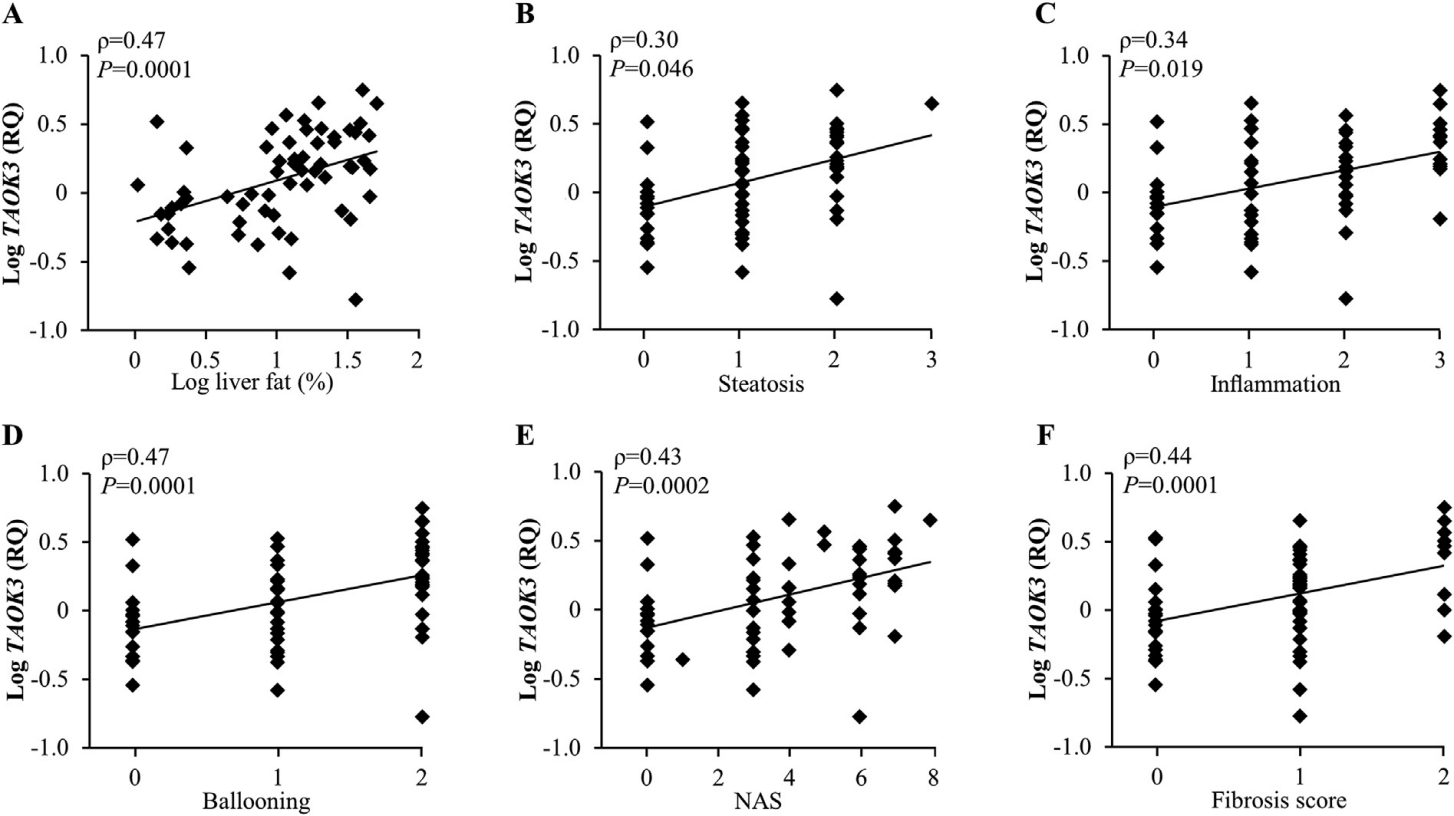
Expression of *TAOK3* mRNA in human liver biopsies is significantly and positively correlated with the severity of NAFLD. (A) Correlation between the hepatic fat content measured by magnetic resonance spectroscopy (^1^H-MRS) and the *TAOK3* mRNA expression assessed in human liver biopsies by qRT-PCR. (B–F) Correlation between the *TAOK3* mRNA expression determined in human liver biopsies by qRT-PCR and three individual histologic lesions of NAS (*i.e.*, steatosis, lobular inflammation, and hepatocellular ballooning; B–D), the total NAS (E), and the fibrosis score (F). RQ, relative quantification.

Next, we evaluated the association between hepatic *TAOK3* mRNA and the widely used histological score of NAFLD severity – NAS. We found a positive correlation between the *TAOK3* levels and all three individual components of the NAS (*i.e*., liver steatosis, lobular inflammation, and hepatocellular ballooning) as well as total NAS (Figure 1B–E). Notably, subjects with NAS ≥5, which defines definite NASH (*n* = 24), had a 1.8 ± 0.2-fold increase in the *TAOK3* expression compared with subjects with NAS ≤4, which indicates simple steatosis or borderline NASH (*n* = 38; *P* = 0.008). Importantly, a significant positive correlation was also detected between *TAOK3* abundance and the histological fibrosis score (Figure 1F).

It has been well established that NAFLD and T2D coexist and act synergistically to cause negative outcomes in clinical practice [1]. Based on this evidence, an additional correlation analysis was performed in a subset of 24 participants who had been diagnosed with T2D. Significant positive correlations were found between the hepatic *TAOK3* mRNA levels and the liver fat content, as well as NAS and the fibrosis score, even in this cohort (Supplementary Figure S2).

Recently, NASH has been recognized as a major catalyst for HCC, which is one of the most fatal and fastest-growing cancers [[40], [41], [42]]. Interestingly, the microarray data analysis of the large cohort of HCC patients available at the GEO database (n = 214) demonstrated that the *TAOK3* gene expression was significantly higher in HCC tumors than in the adjacent non-tumor liver tissue (*P* < 0.0001; GSE14520). TAOK3 protein abundance was also enhanced in frequently used human HCC cell lines (poorly differentiated SNU-475 and well-differentiated Hep3B) *versus* non-tumor IHHs (Supplementary Figure S3). Furthermore, TAOK3 protein levels were increased in the livers of mice with HCC induced by the administration of diethylnitrosamine (DEN) combined with a high-fat diet or treatment with carbon tetrachloride (CCl_4_) combined with a choline-deficient l-amino-acid-defined (CDAA) diet, when compared with those observed in the samples collected from healthy chow-fed control mice (Supplementary Figure S4).

### 3.2 TAOK3 decorates intrahepatocellular lipid droplets

Our previous studies have identified TAOK3 by proteomic analysis performed on the lipid droplet fraction from steatotic livers of high-fat-diet-fed mice [14,15]. Importantly, the proteins isolated by this approach do not necessarily represent *bona fide* lipid droplet proteins residing primarily or exclusively on the droplets, since the method fails to effectively deplete the membrane-bound cellular organelles that closely associate with lipid droplets, including the ER, mitochondria, peroxisomes, and endosomes [43,44]. Here, we further investigated the subcellular localization of TAOK3 in human hepatocytes and mouse liver using immunofluorescence microscopy. We found that TAOK3 protein was closely associated with lipid droplets, visualized by ADRP (also known as adipophilin or perilipin-2) staining, both in cultured human hepatocytes and in mouse liver sections (Figure 2A). Interestingly, we also found that hepatic TAOK3 protein abundance was increased 3.8 ± 0.6-fold in mice in response to the challenge with a high-fat diet (Figure 2B). Notably, we detected the *TAOK3* transcript in all the human, mouse, and rat tissues analyzed (Figure 2C), which is consistent with the findings of previous studies describing the ubiquitous expression pattern of human *TAOK3* [20,45]. The high *TAOK3* levels observed in organs that do not store a significant amount of lipids, particularly in the human lungs, suggest that TAOK3 may display a different subcellular localization pattern in extrahepatic tissues.

**Figure 2:**
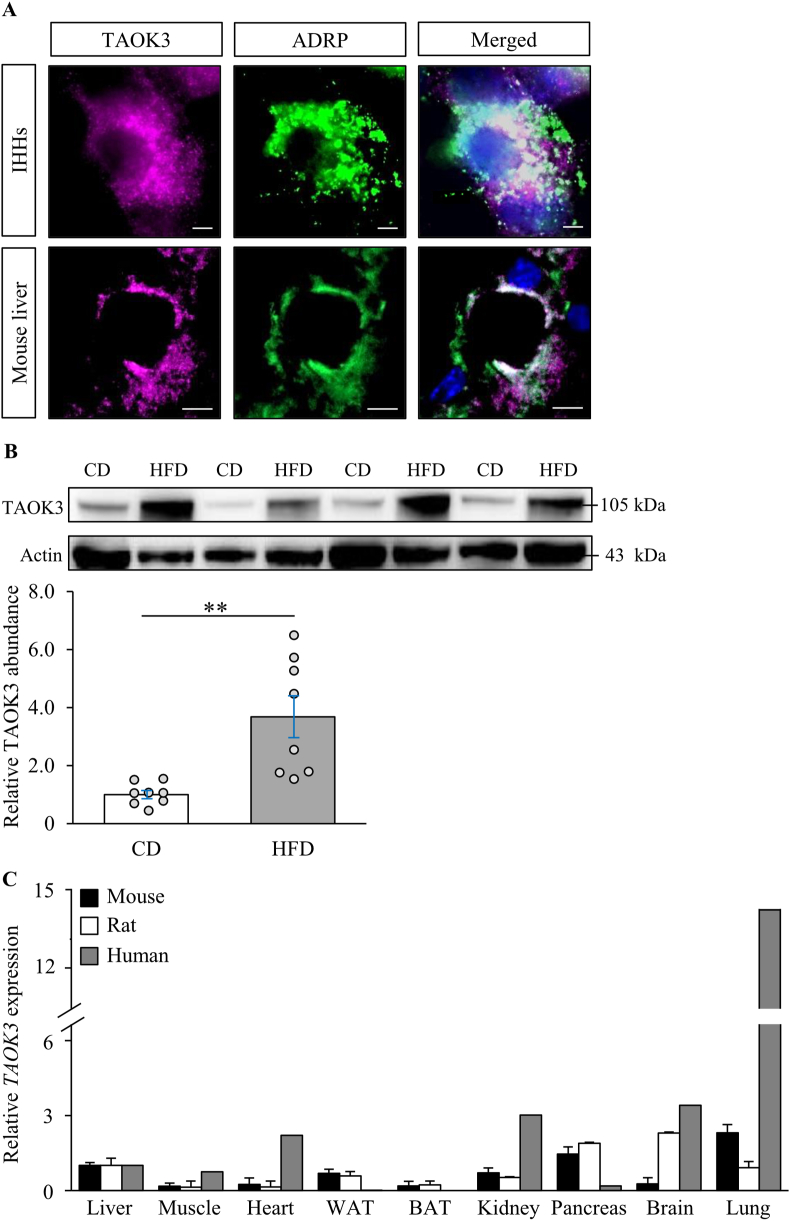
TAOK3, expressed in a broad range of tissues, associates with intracellular lipid droplets in the liver. (A) Representative immunofluorescence images of IHHs challenged with oleic acid and liver sections from high-fat-fed mice, double-stained with antibodies for TAOK3 (violet) and ADRP (green); the merged image shows colocalization in white with nuclei stained with DAPI (blue). The scale bars at the top and bottom represent 10 μm and 15 μm, respectively. (B) Liver lysates from mice fed with a high-fat diet (60 kcal% fat; D12492; Research Diets, New Brunswick, NJ) for 20 weeks, and from age-matched chow-fed controls, were assessed by Western blot. Protein levels were analyzed by densitometry; the representative Western blots are shown, with actin used as a loading control. Data are represented as mean ± SEM from 8 mice per group. (C) The relative *TAOK3* mRNA expression was assessed by qRT-PCR in mouse, rat, and human tissues. The transcript level of TAOK3 in the liver of each species is set to 1. Mouse and rat data are represented as mean ± SEM from 3 to 6 animals. Human MTC Panel I (Takara, Kyoto, Japan) was analyzed for human expression data. CD, chow-diet; HFD, high-fat diet; BAT, brown adipose tissue; WAT, white adipose tissue. ∗∗*P* < 0.01.

### 3.3 TAOK3 controls intrahepatocellular lipid partitioning

To further examine the causative relationship between hepatic TAOK3 levels and liver steatosis, we characterized the effect of modifying the TAOK3 abundance on lipid metabolism in human hepatocytes. For TAOK3 overexpression, we transfected IHHs with human *MYC*-tagged *TAOK3* expression plasmid or an empty control plasmid (Supplementary Figure S5). In parallel to the assays performed under basal culture conditions, we also treated the cells with oleic acid to replicate the environment in high-risk subjects (Supplementary Figure S5). IHHs transfected with *MYC*-tagged *TAOK3* displayed a robust increase in TAOK3 mRNA and protein abundance compared with cells transfected with vector control (Figure 3A and B). Immunostainings with anti-MYC antibody also demonstrated a high transfection efficacy of >90% in IHHs transfected with *MYC-TAOK3* expression plasmid (Supplementary Figure S6). First, we stained the transfected cells with Bodipy 493/503 for visualizing neutral lipids within the lipid droplets. We found a significant increase in the Bodipy-stained area in TAOK3-overexpressing IHHs (Figure 3C and D). Despite this marked change in the cellular lipid accumulation, TAOK3 overexpression had no impact on the membrane lipid composition (Supplementary Figure S7A). Mechanistically, we detected lower mitochondrial content and activity, measured by the staining of MitoTracker Green and Red, respectively, in IHHs transfected with *TAOK3* expression plasmid, in parallel with the reduced β-oxidation rate (Figure 3C–E; Supplementary Figure S8A). Noteworthily, the pronounced decline in MitoTracker Green- and Red-positive areas in TAOK3-overexpressing cells (about 1.5- to 3-fold reduction detected both under basal conditions and after exposing the cells to oleic acid) was accompanied by a rather modest decrease in fatty acid oxidation (1.14 ± 0.16-fold reduction found only in cells treated with oleic acid). The reason for this discrepancy remains unknown. TAOK3 overexpression also significantly decreased the secretion of *de novo*-synthesized TAG into the media (Figure 3F). Reciprocally, there was a 2-fold increase in the incorporation of medium-derived glucose into intracellular TAG in IHHs transfected with the *TAOK3* expression plasmid *versus* vector control, with no alteration in the fatty acid uptake (Figure 3G and H). The concentrations of phosphohexoses and several amino acids were significantly lower in TAOK3-overexpressing hepatocytes, which likely reflects a shift in the energy metabolism from the utilization of lipids toward carbohydrates and amino acids (Supplementary Figure S9A). Importantly, increased TAOK3 abundance had no effect on the cell viability (Figure 3I).

**Figure 3:**
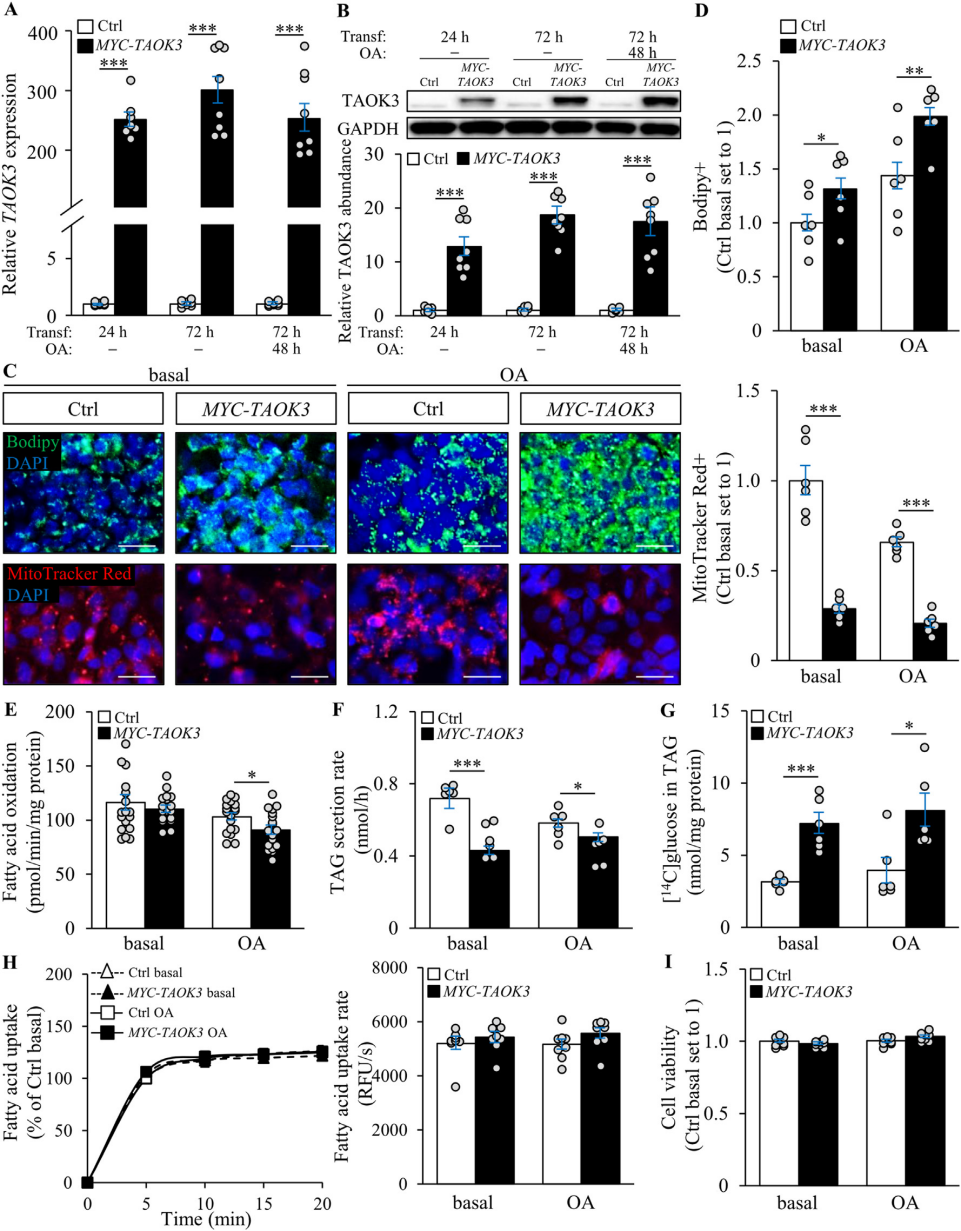
Overexpression of TAOK3 increases lipid accumulation in IHHs. IHHs were transfected with the *MYC*-tagged *TAOK3* expression plasmid or an empty control plasmid. All the assessments were performed under basal culture conditions and after exposing the cells to oleic acid for 48 h. (A–B) TAOK3 mRNA (A) and protein (B) abundance was assessed by qRT-PCR and Western blot, respectively. Protein levels were analyzed by densitometry; the representative Western blots are shown, with glyceraldehyde-3-phosphate dehydrogenase (GAPDH) used as a loading control. (C–D) Representative images of cells stained with Bodipy (green) or MitoTracker Red (red); nuclei stained with DAPI (blue) (C). The scale bars at the top and bottom represent 15 μm and 20 μm, respectively. Quantification of the staining (D). (E) Oxidation of radiolabeled palmitate. (F) Secretion of [^3^H]TAG into the media. (G) TAG synthesis from [^14^C]-labeled glucose. (H) Fatty acid uptake rate. (I) Cell viability. Data are represented as mean ± SEM from 6 to 20 wells per group. Ctrl, control; OA, oleic acid; Transf, transfection. ∗*P* < 0.05, ∗∗*P* < 0.01, ∗∗∗*P* < 0.001.

To study the metabolic effects of TAOK3 silencing in human hepatocytes, we transfected IHHs with *TAOK3*-specific siRNA or with a non-targeting control (NTC) siRNA (Supplementary Figure S10). As expected, TAOK3 mRNA and protein expression was efficiently silenced in cells transfected with *TAOK3* siRNA (Figure 4A and B). In contrast to our observations in TAOK3-overexpressing cells, the Bodipy-positive area was significantly lower in TAOK3-deficient IHHs (Figure 4C and D). Noteworthily, this difference in the cellular lipid deposition did not affect the membrane lipid composition (Supplementary Figure S7B). We also found that TAOK3 knockdown in IHHs enhanced mitochondrial biogenesis, fatty acid oxidation, and the rate of TAG secretion (Figure 4C–F; Supplementary Figure S8B). In contrast, both *de novo* lipogenesis and fatty acid influx were suppressed in IHHs where TAOK3 was silenced (Figure 4G and H). Notably, the significant increase in β-oxidation and the reduction in free fatty acid uptake were detected only in TAOK3-deficient cells incubated with oleic acid (Figure 4E and H). The concentration of phosphohexoses and the levels of most of the amino acids were unaffected by TAOK3 knockdown (Supplementary Figure S9B). We observed no difference in cell viability in IHHs transfected with *TAOK3* siRNA *versus* NTC siRNA (Figure 4I).

**Figure 4:**
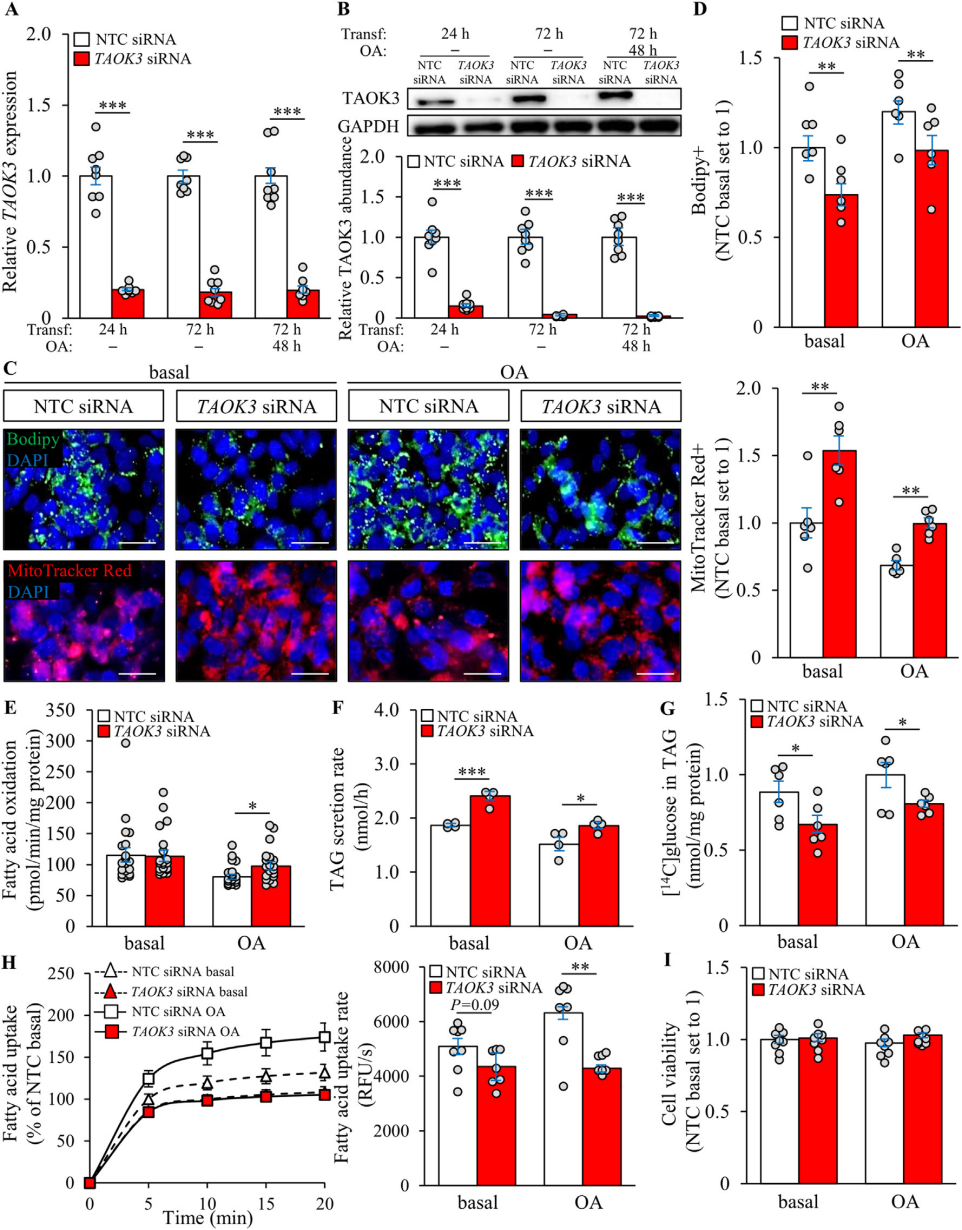
Silencing of TAOK3 decreases lipid accumulation in IHHs. IHHs were transfected with *TAOK3* siRNA or NTC siRNA. All the assessments were performed under basal culture conditions and after exposing cells to oleic acid for 48 h. (A–B) TAOK3 mRNA (A) and protein (B) abundance was assessed by qRT-PCR and Western blot, respectively. Protein levels were analyzed by densitometry; the representative Western blots are shown, with GAPDH used as a loading control. (C–D) Representative images of cells stained with Bodipy (green) or MitoTracker Red (red); nuclei stained with DAPI (blue) (C). The scale bars at the top and bottom represent 15 μm and 20 μm, respectively. Quantification of the staining (D). (E) Oxidation of radiolabeled palmitate. (F) Secretion of [^3^H]TAG into the media. (G) TAG synthesis from [^14^C]-labeled glucose. (H) Fatty acid uptake rate. (I) Cell viability. Data are represented as mean ± SEM from 6 to 20 wells per group. OA, oleic acid; Transf, transfection. ∗*P* < 0.05, ∗∗*P* < 0.01, ∗∗∗*P* < 0.001.

Based on the close association of TAOK3 with intrahepatocellular lipid droplets, we next tested the hypothesis that TAOK3 controls lipid mobilization from the droplets by regulating canonical lipolysis. In this process, a series of lipases binding to the lipid droplet surface sequentially catabolize TAG into free fatty acids, which are subsequently targeted to mitochondria for β-oxidation or directed to the ER/Golgi for the synthesis and secretion of very low-density lipoprotein (VLDL)-TAG [46]. Indeed, we detected significantly reduced or enhanced lipolysis rate (assay measuring TAG hydrolase activity using triolein as the substrate [47]) in IHHs treated with oleic acid, when TAOK3 was overexpressed or knocked down, respectively (Figure 5A and B).

**Figure 5:**
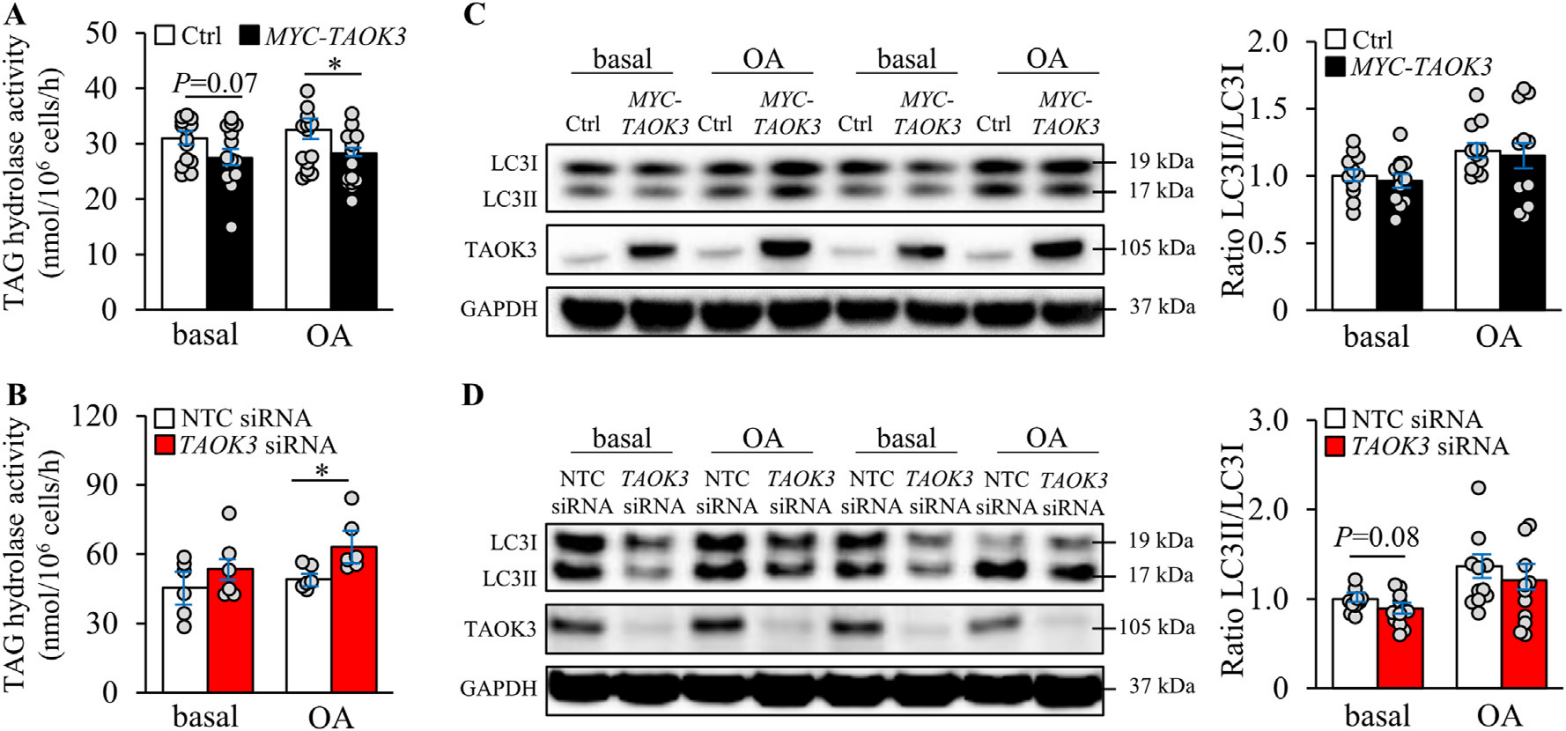
Modifying the abundance of TAOK3 in IHHs has an impact on lipolysis but not on autophagy. IHHs were transfected with the *MYC*-tagged *TAOK3* expression plasmid or an empty control plasmid (A,C) or with *TAOK3* siRNA or NTC siRNA (B,D). The assessments were performed under basal culture conditions and after exposing the cells to oleic acid for 48 h. (A,B) TAG hydrolase activity was measured using [^3^H]triolein as the substrate. (C,D) Cell lysates were analyzed by Western blot using antibodies specific for LC3 or TAOK3. Protein levels were analyzed by densitometry; the representative Western blots are shown, with GAPDH used as a loading control. Data are represented as mean ± SEM from 6 to 11 wells per group. Ctrl, control; OA, oleic acid. ∗*P* < 0.05.

As an alternative to lipolysis, lipids can be mobilized from the droplets by selective autophagy (also called lipophagy) [48]. To test the hypothesis that TAOK3 regulates lipid catabolism *via* autophagy, we measured the conversion of LC3-I to LC3-II, which is considered to be a key marker of autophagic flux. However, we did not detect any alterations in the LC3-II to LC3-I ratio in IHHs where TAOK3 was overexpressed or silenced (Figure 5C and D).

### 3.4 TAOK3 regulates oxidative and endoplasmic reticulum stress in human hepatocytes

Accumulation of excessive lipids in hepatocytes is known to aggravate oxidative and ER stress, which in turn fuels liver inflammation, fibrosis, and apoptosis, thus triggering disease progression from simple steatosis toward NASH [49]. Consistent with this evidence, we observed that exacerbated lipid storage in TAOK3-overexpressing IHHs was accompanied by significantly increased oxidative stress as evidenced by the higher abundance of superoxide radicals (O^•−^) measured by DHE staining, elevated oxidative DNA damage quantified by immunostaining for 8-oxoG, and increased deposition of lipid peroxidation products and oxidized phospholipids detected by immunostaining for 4-HNE and E06, respectively (Figure 6A; Supplementary Figure S11A). Furthermore, we found enhanced immunostaining for KDEL (a signal motif for ER retrieval) and CHOP (an indicator of ER stress-induced cell death) in IHHs transfected with the *MYC*-tagged *TAOK3* expression plasmid *versus* vector control (Figure 6A; Supplementary Figure S11A). In contrast, TAOK3-deficient IHHs were significantly protected against oxidative damage as indicated by the suppressed abundance of DHE, 8-oxoG, 4-HNE, and E06 as well as ER stress as measured by the lower levels of KDEL and CHOP (Figure 7A; Supplementary Figure S12A). Notably, several mRNA indicators of oxidative and ER stress, as well as pro-apoptotic markers, were significantly decreased in IHHs transfected with *TAOK3* siRNA *versus* NTC siRNA (Figure 7B; Supplementary Figure S12B), while no coordinated increase was detected in cells transfected with *MYC*-tagged *TAOK3* expression plasmid *versus* vector control (Figure 6B; Supplementary Figure S11B). At present, we cannot explain the mechanisms by which TAOK3 regulates transcription. However, a lower transcriptional response in TOAK3-overexpressing IHHs suggests a possibility that different critical thresholds for TAOK3 may exist in hepatocytes.

**Figure 6:**
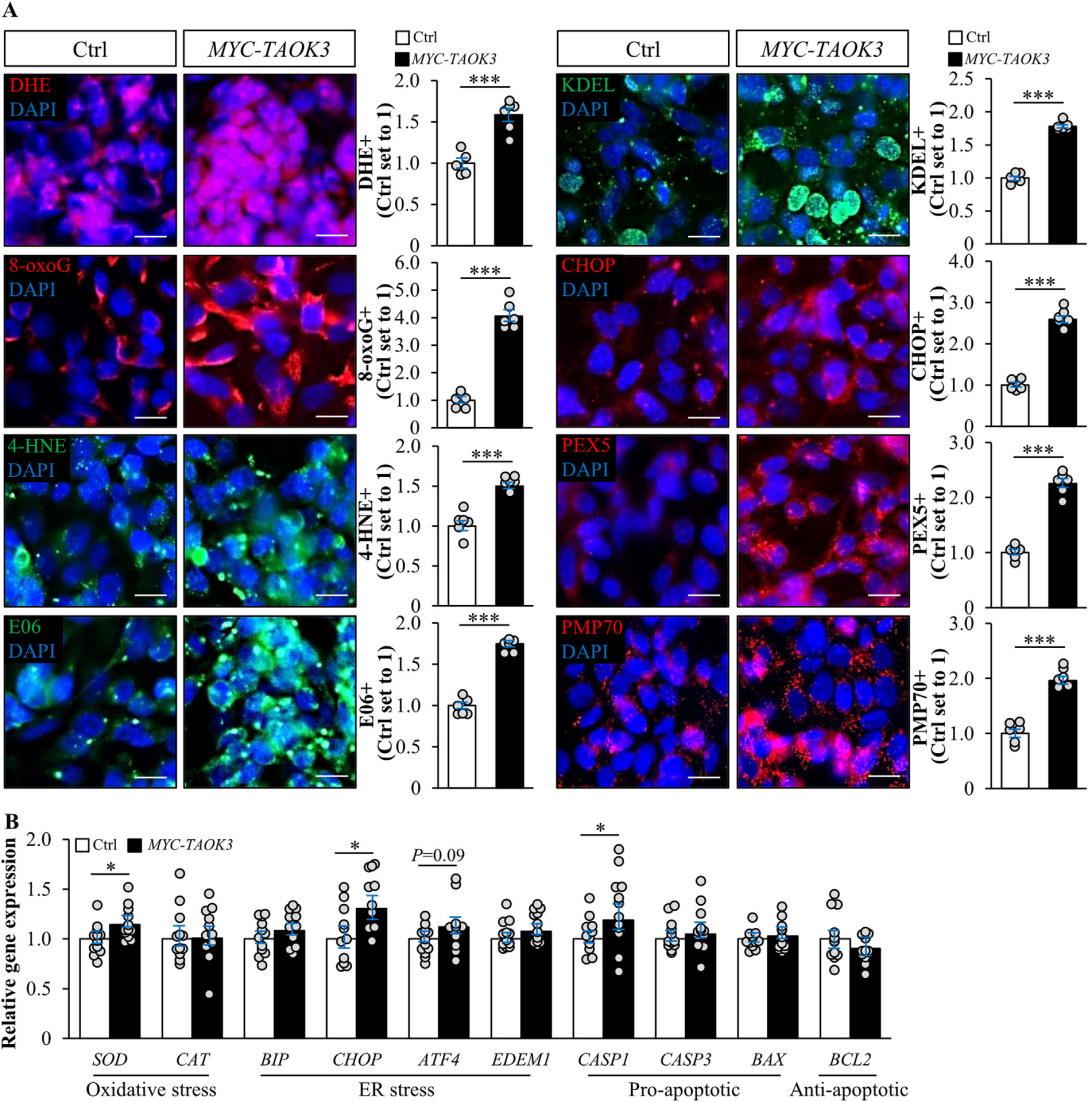
Overexpression of TAOK3 exacerbates oxidative and ER stress in IHHs. IHHs were transfected with the *MYC*-tagged *TAOK3* expression plasmid or an empty control plasmid and challenged with oleic acid for 48 h. (A) Representative images of cells stained with DHE (red) or processed for immunofluorescence with anti-8-oxoG (red), anti-4-HNE (green), anti-E06 (green), anti-KDEL (green), anti-CHOP (red), anti-PEX5 (red), or anti-PMP70 (red) antibodies; nuclei stained with DAPI (blue). The scale bars represent 15 μm. Quantification of the staining. (B) Relative mRNA expression of selected genes controlling oxidative and ER stress as well as apoptosis was assessed by qRT-PCR. Data are represented as mean ± SEM from 6 to 12 wells per group. Ctrl, control. ∗*P* < 0.05, ∗∗∗*P* < 0.001.

**Figure 7:**
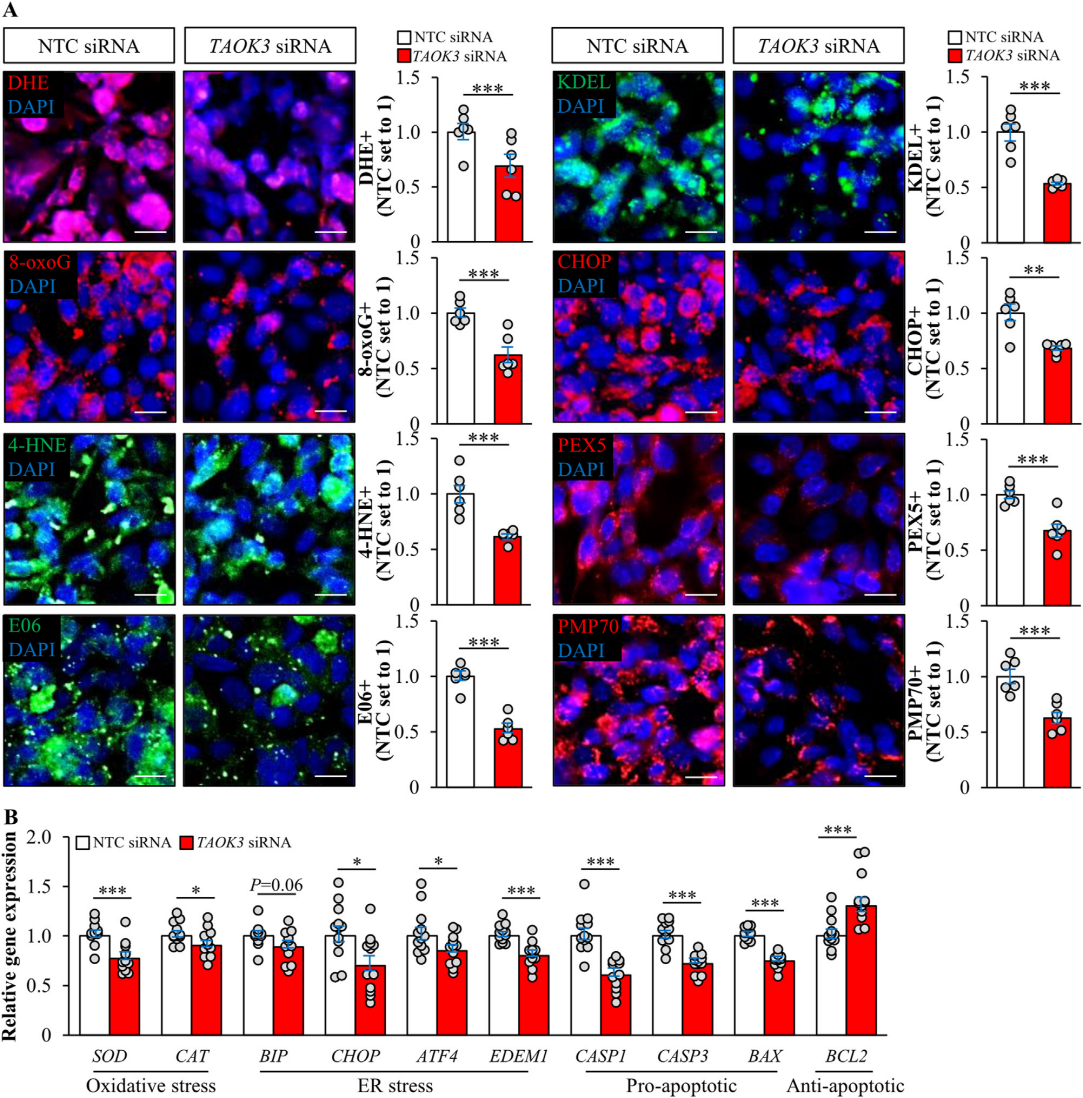
Silencing of TAOK3 suppresses oxidative and ER stress in IHHs. IHHs were transfected with *TAOK3* siRNA or NTC siRNA and challenged with oleic acid for 48 h. (A) Representative images of cells stained with DHE (red) or processed for immunofluorescence with anti-8-oxoG (red), anti-4-HNE (green), anti-E06 (green), anti-KDEL (green), anti-CHOP (red), anti-PEX5 (red), or anti-PMP70 (red) antibodies; nuclei stained with DAPI (blue). The scale bars represent 15 μm. Quantification of the staining. (B) Relative mRNA expression of selected genes controlling oxidative and ER stress as well as apoptosis was assessed by qRT-PCR. Data are represented as mean ± SEM from 6 to 12 wells per group. ∗*P* < 0.05, ∗∗*P* < 0.01, ∗∗∗*P* < 0.001.

Interestingly, we found increased or diminished peroxisomal activity in IHHs in which TAOK3 was overexpressed or silenced, respectively, as shown by alternations in immunostaining for the peroxisome biogenesis marker PEX5 and the peroxisomal membrane protein PMP70 (Figure 6, Figure 7A; Supplementary Figures S11A and S12A).

### 3.5 TAOK3 deficiency inhibits JNK signaling and suppresses the STE20-type kinase STK25 abundance

Silencing of TAOK3 has previously been implicated both in the inhibition (human mesenchymal stromal cells) and activation (HeLa cervical carcinoma cells) of c-Jun-N terminal kinase (JNK) signaling [50,51]; however, the possible role of TAOK3 in the regulation of the JNK pathway has not been studied in hepatocytes. Here we found that the phosphorylation of JNK was significantly lower in IHHs transfected with *TAOK3* siRNA compared with NTC siRNA, both under basal culture conditions and after exposing the cells to oleic acid (Figure 8A). TAO kinases have also been implicated in the regulation of p38 MAPK and Hippo pathways [45]. We did not detect any significant alterations in the phosphorylation of Yes-associated protein (YAP), the major effector of Hippo signaling, in TAOK3-deficient hepatocytes (Figure 8A) while phosphorylation of p38 remained below the level of quantification in IHHs transfected with both *TAOK3* siRNA and NTC siRNA (data not shown).

**Figure 8:**
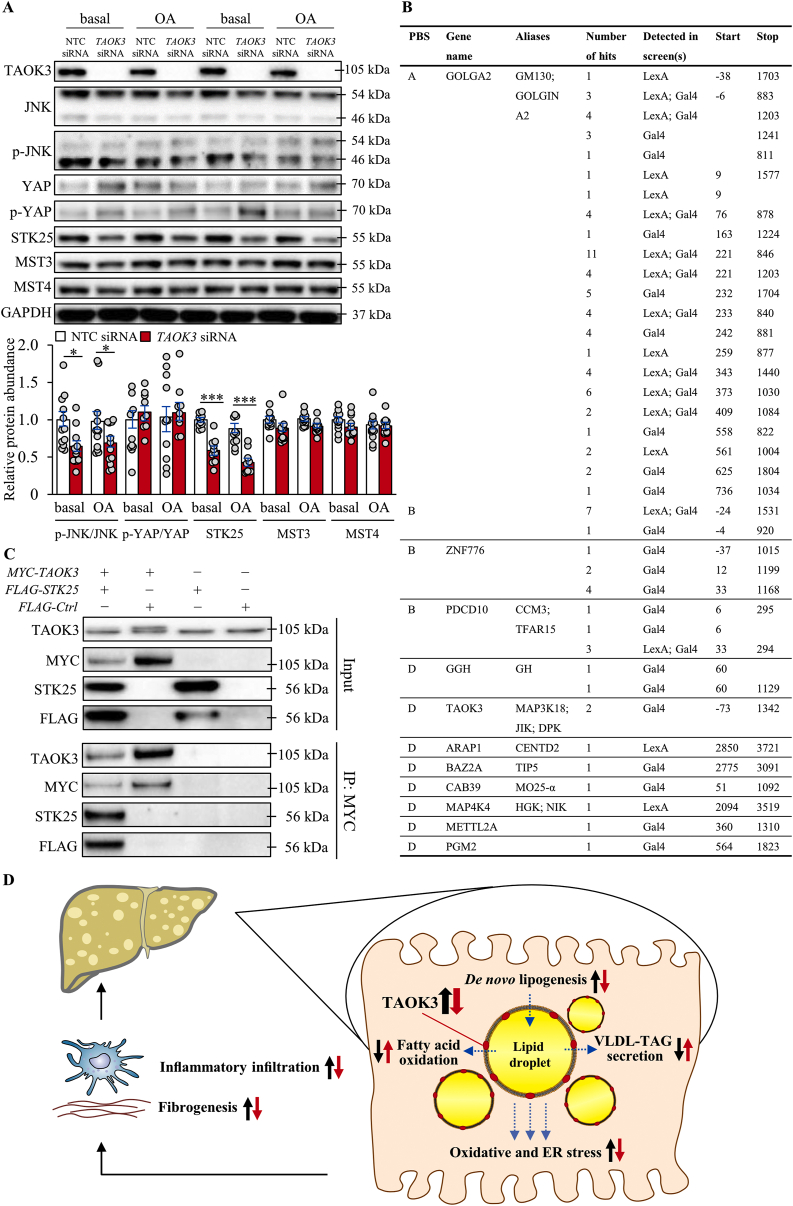
TAOK3 affects JNK activation and interacts with STK25. (A) IHHs were transfected with *TAOK3* siRNA or NTC siRNA. The assessments were performed under basal culture conditions and after exposing the cells to oleic acid for 48 h. The cell lysates were analyzed by Western blot using antibodies specific for JNK, phospho-JNK (Thr^183^/Tyr^185^), YAP, phospho-YAP (Ser^127^), STK25, MST3, MST4, or TAOK3. Protein levels were analyzed by densitometry; the representative Western blots are shown with GAPDH used as a loading control. Data are represented as mean ± SEM from 11–12 wells per group. (B) Y2H screening identifies TAOK3 as a binding partner of STK25. Candidate interactors of STK25 with PBS scores of A–D detected in a Y2H screen using STK25 as bait are shown: A, very high confidence in the interaction; B, high confidence in the interaction; C, good confidence in the interaction; D, moderate confidence in the interaction. (C) Co-immunoprecipitation of TAOK3 and STK25 was performed from protein extracts of IHHs transfected with *MYC-TAOK3*, *FLAG-STK25,* and/or *FLAG-Control* plasmid. The starting material as well as protein immunoprecipitated using anti-MYC antibodies were analyzed by Western blot using antibodies specific for TAOK3, MYC, STK25, or FLAG; the representative Western blots are shown. (D) A working model of the function of TAOK3 in regulating liver lipotoxicity. Overexpression of TAOK3 in hepatocytes stimulates lipid droplet anabolism through increased TAG synthesis and inhibits lipid droplet catabolism through suppressed β-oxidation and VLDL-TAG secretion, augmenting oxidative and ER stress. Although not investigated in our study, these alterations in TAOK3-overexpressing cells may subsequently lead to hepatic inflammation and fibrogenesis. The opposite changes in intrahepatocellular lipid storage and oxidative/ER stress are observed when TAOK3 is knocked down. Ctrl, control; IP, immunoprecipitated material; OA, oleic acid. ∗*P* < 0.05, ∗∗∗*P* < 0.001.

Our recent studies have revealed that depletion of STE20 kinases STK25, MST3, or MST4 protects against intrahepatocellular lipid accumulation [[9], [10], [11], [12], [13], [14], [15], [16], [17]], similarly to the silencing of TAOK3. In the light of this evidence, we next investigated whether the abundance of STK25, MST3, or MST4 protein was altered in TAOK3-deficient hepatocytes. We found that STK25 levels were significantly lower in IHHs transfected with *TAOK3* siRNA *versus* NTC siRNA, while no difference was detected in MST3 or MST4 (Figure 8A). TAOK3 appears to regulate STK25 abundance at the level of protein expression and/or stability, since the *STK2*5 mRNA content was not affected (Supplementary Figure S13).

To identify the novel interacting proteins of STK25, we performed a genome-wide Y2H screening of a primary human hepatocyte cDNA library using full-length human STK25 fused to the LexA or Gal4 DNA-binding domain as bait. The screening revealed a total of 11 potential interaction partners of STK25 (Figure 8B). Interestingly, TAOK3 was identified as the binding partner for STK25 when using the Gal4-STK25 as bait, which is considered to have higher sensitivity for detecting weak interactions compared with LexA fusions. The prey fragment of TAOK3 that interacted with STK25 (amino acids 1 to 447) harbors the N-terminal kinase domain and the serine-rich domain of unknown function but excludes the C-terminal coiled-coil regions implicated in the formation of TAOK3 oligomers [45,52]. Notably, we recovered clones for the previously identified STK25-interactors GOLGA2 (also known as GM130 or GOLGIN A2) [53,54], PDCD10 (also known as CCM3 or TFAR15) [55,56], and CAB39 (also known as MO25-α) [57], verifying the high quality of the screen.

To confirm the direct interaction between TAOK3 and STK25, we next transfected IHHs with plasmids encoding *MYC-TAOK3* and *FLAG-STK25*. Using anti-MYC immunoprecipitation, we were able to verify the direct binding between full-length TAOK3 and STK25 (Figure 8C). Furthermore, by immunoprecipitation of the protein extracts from IHHs transfected with the truncated versions of the *MYC*-tagged *TAOK3* expression plasmid, we confirmed that the N-terminal fragment of TAOK3-containing kinase and serine-rich domains, but not the C-terminal fragment of the TAOK3-containing coiled-coil regions, interacted with STK25 (Supplementary Figure S14).

## 4 Discussion

Deciphering the molecular mechanisms underlying the onset and progression of NAFLD is essential to discover effective therapeutic strategies for its prevention and treatment. This study provides the first evidence for a possible role of the liver lipid droplet-binding STE20-type kinase TAOK3 in NAFLD pathogenesis.

The primary driver in NAFLD is an imbalance in hepatic lipid metabolism, which leads to the accumulation of intrahepatocellular fat, which then fuels oxidative and ER stress, local inflammation, cell damage, and subsequent fibrogenesis in the liver [49,58]. Importantly, we found that the overexpression of TAOK3 in human hepatocytes enhanced *de novo* lipogenesis (*i.e*., input) and reduced β-oxidation and TAG secretion (*i.e*., output) resulting in aggravated fat storage within lipid droplets, and the opposite effect was observed in TAOK3-deficient hepatocytes (Figure 8D). Consistently, oxidative and ER stress was substantially exacerbated or suppressed in human hepatocytes where TAOK3 was overexpressed or silenced, respectively (Figure 8D). In line with these results, we found that *TAOK3* expression in human liver biopsies was positively correlated with hepatic steatosis measured by magnetic resonance spectroscopy as well as histological scoring. Furthermore, we detected a significant positive correlation between *TAOK3* levels in human liver biopsies and hepatic inflammation, cell damage, and fibrosis scores. Together, these results suggest that TAOK3 regulates a critical node governing hepatocellular lipid homeostasis and that TAOK3 antagonism could mitigate NAFLD initiation as well as disease progression toward NASH.

The main technical limitation of this study is our inability to delineate the primary *versus* the secondary changes in response to the modifications in the abundance of TAOK3 in hepatocytes. For example, alterations in oxidative and ER stress can be caused by increased or decreased lipid storage in cells where TAOK3 is overexpressed or silenced, respectively; however, TAOK3 may also have a direct impact on the metabolic stress response.

Notably, we found that TAOK3 displays a very distinct subcellular localization pattern in both human and rodent hepatocytes and is associated with intracellular lipid droplets. We also demonstrate that TAOK3 protein abundance was increased severalfold in mouse liver in response to a challenge with a high-fat diet. This observation is in line with the recent evidence showing that the hepatic lipid droplet proteome is highly dynamic, being influenced by dietary composition as well as the liver metabolic status [46].

Interestingly, we observed that the silencing of TAOK3 suppressed JNK signaling in hepatocytes. Recent reports have revealed that decreased mitochondrial fat oxidation in liver steatosis and NASH is initiated by the activation of JNK, which can phosphorylate mitochondrial proteins [59]. Reciprocally, hepatocyte-specific inhibition of the JNK pathway has been demonstrated to enlarge the mitochondria and increase mitochondrial β-oxidation, protecting the mice from liver steatosis [60]. Thus, decreased JNK phosphorylation may have contributed to a significant increase in the mitochondrial activity observed in TAOK3-deficient hepatocytes.

We found that TAOK3 forms a complex in human hepatocytes with another kinase of the STE20 family – STK25. Furthermore, the silencing of TAOK3 in hepatocytes significantly reduced the protein abundance of STK25. This finding is relevant in light of our recent studies demonstrating that depletion of STK25, similarly to TAOK3 deficiency, suppresses intracellular fat accumulation in hepatocytes by shifting the metabolic balance from lipid synthesis toward lipid utilization [[9], [10], [11], [12], [13], [14]]. Thus, the regulation of STK25 levels may constitute a part of the mechanism of action of TAOK3 in the liver.

In addition to TAOK3 and STK25, the STE20-type kinases MST3 and MST4 have been associated with molecular pathogenesis of NAFLD, as evidenced by (i) protein localization to the surface of intrahepatocellular lipid droplets, (ii) inhibition of the target suppressing liver lipid accumulation in mouse models and/or cultured human hepatocytes, (iii) a significant correlation between the hepatic expression levels of the kinase and the severity of NAFLD in humans [[15], [16], [17]]. At this juncture, we are only in the infancy of mechanistic understanding of the role of STE20 kinases in liver lipid droplet dynamics and NAFLD; however, this family emerges as an important node regulating hepatic lipotoxicity, warranting further investigations in basic biology as well as clinical implications.

NAFLD is currently the most rapidly increasing cause of liver-related morbidity and mortality, with a substantial health economic burden and no approved therapy [1]. The primary insult in NAFLD is excessive fat accumulation within hepatic lipid droplets and, therefore, the therapeutic strategies against NAFLD may include protection against and/or resolution of liver steatosis, analogous to the concept of lowering lipids to prevent atherosclerosis. Our study emphasizes the importance of lipid droplet-binding proteins in the control of intrahepatocellular fat storage and highlights the inhibition of lipid droplet decorating STE20-type kinase TAOK3 as a potential NAFLD therapy *via* antagonizing hepatic steatosis.

## Author contributions

Y.X. generated the bulk of the results. M.C., E.C., S.K.A., and S.S. contributed to the research data. M.H. and R.P. performed lipidomics and metabolomics analysies, respectively. H.-U.M. provided expertise and contributed to the discussion. M.B. carried out qRT-PCR in human liver biopsies. M.M. directed the project, designed the study, interpreted the data, and wrote the manuscript. All the authors revised the article critically for important intellectual content and approved the final version of the article to be published.

## Grant support

This work was supported by grants from the 10.13039/501100004359Swedish Research Council, the West Sweden Avtal om Läkarutbildning och Forskning (ALF) Program, the 10.13039/501100009708Novo Nordisk Foundation, the 10.13039/501100003793Swedish Heart-Lung Foundation, the Swedish Diabetes Foundation, the Å. Wiberg Foundation, the Adlerbert Research Foundation, the I. Hultman Foundation, the F. Neubergh Foundation, the Prof. N. Svartz Foundation, the L. and J. Grönberg Foundation, the W. and M. Lundgren Foundation, and the I.-B. and A. Lundberg Research Foundation.

## Figures and Tables

**Figs1:**
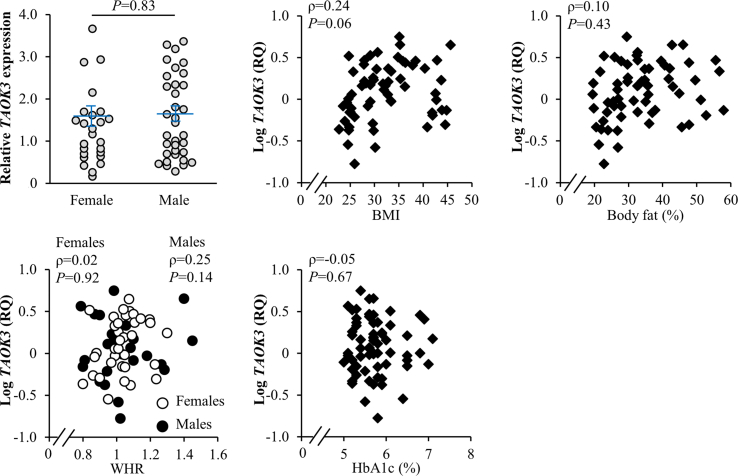


**Figs2:**
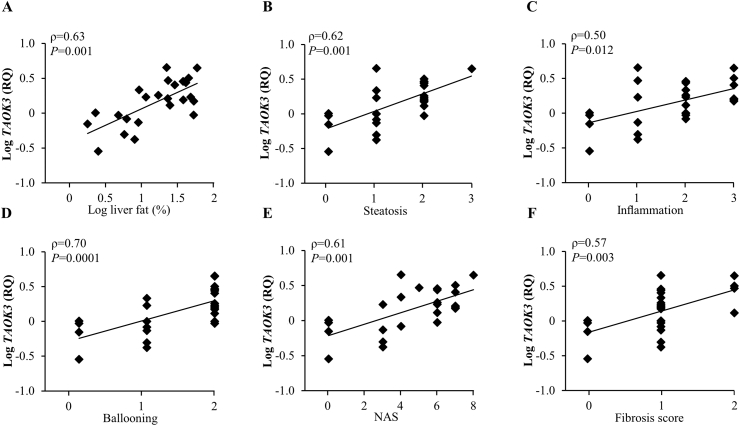


**Figs3:**
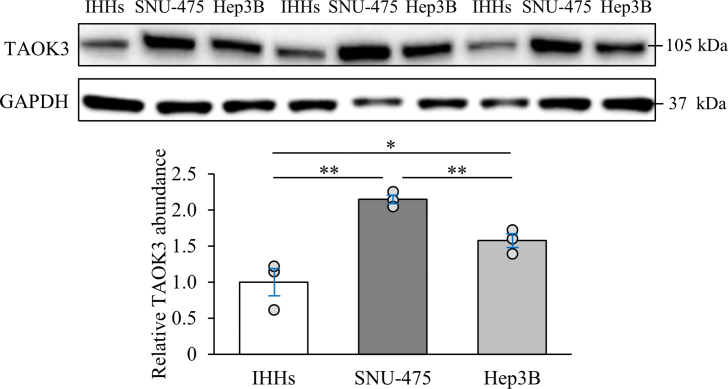


**Figs4:**
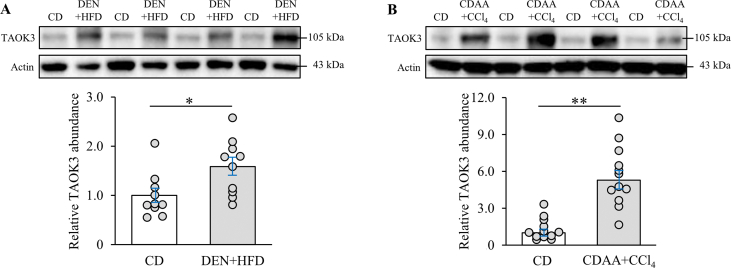


**Figs5:**
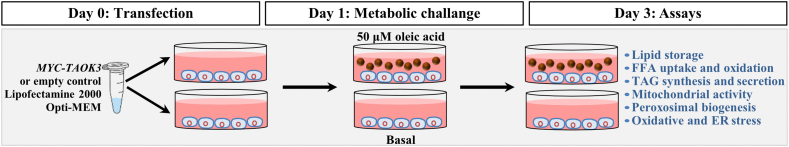


**Figs6:**
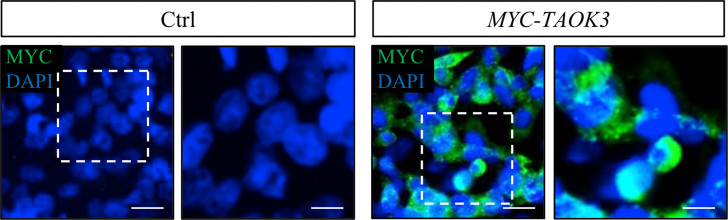


**Figs7:**
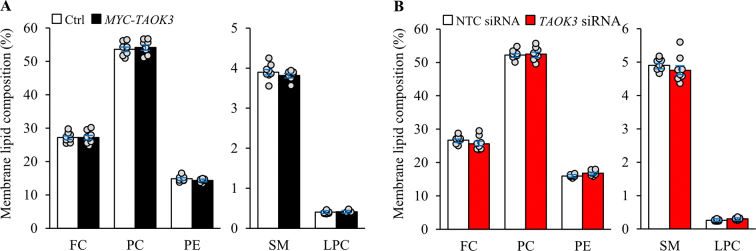


**Figs8:**
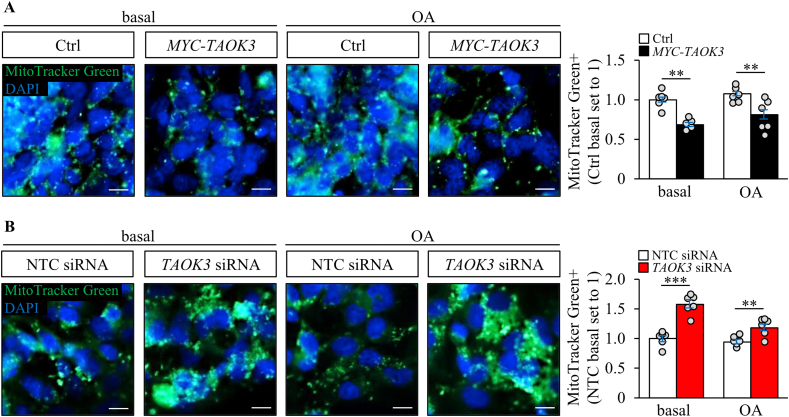


**Figs9:**
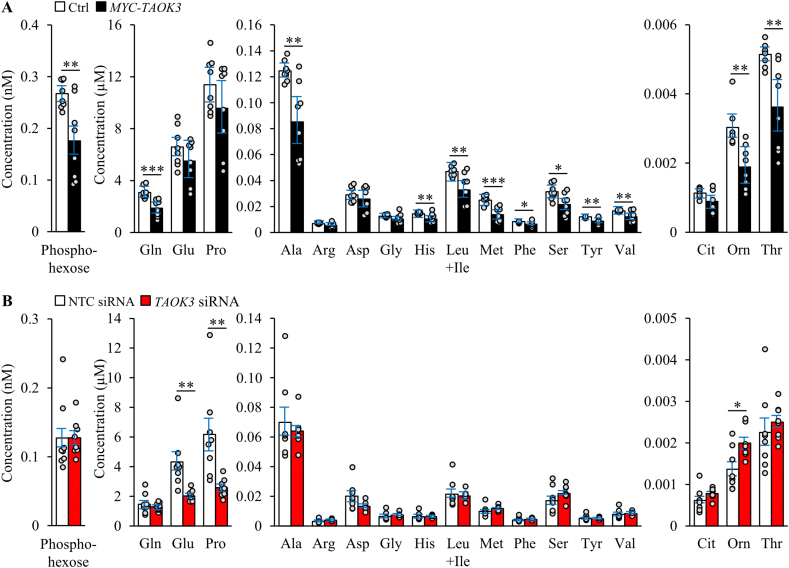


**Figs10:**
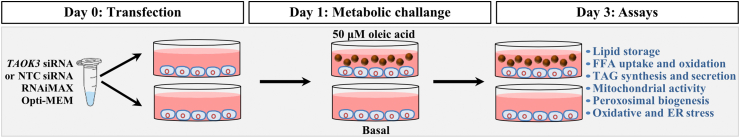


**Figs11:**
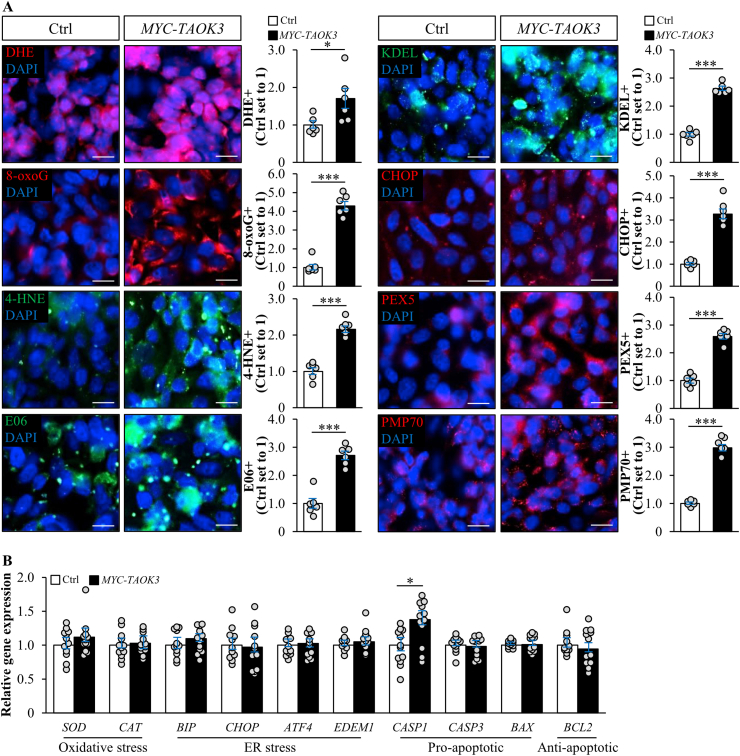


**Figs12:**
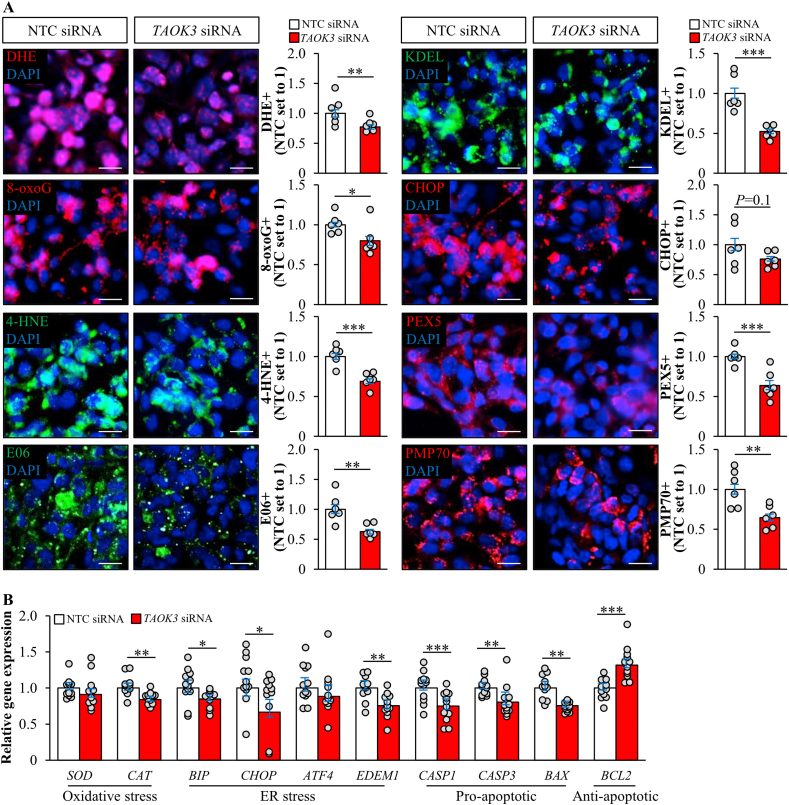


**Figs13:**
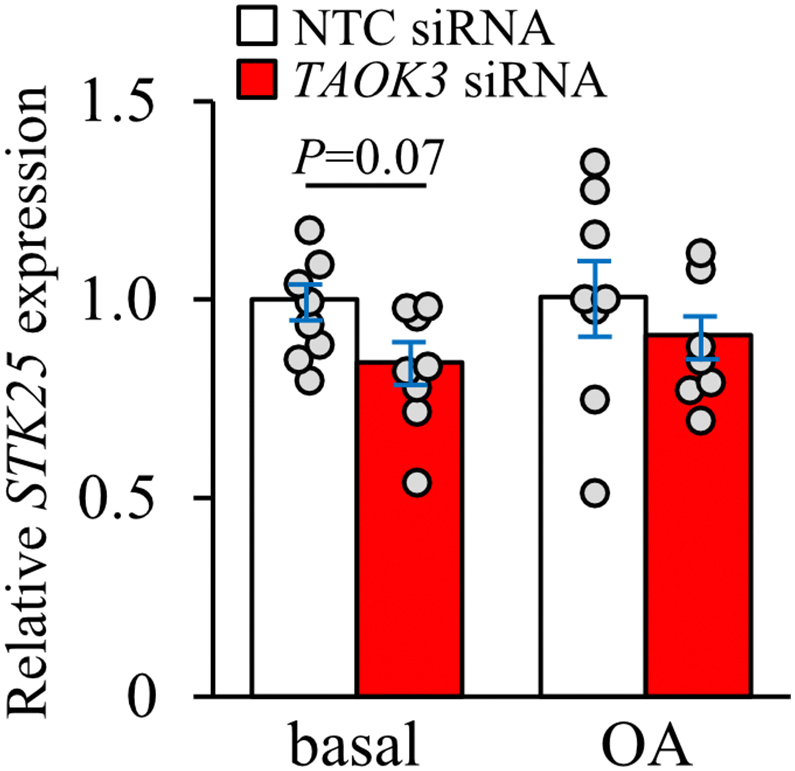


**Figs14:**