# Supplementary material for: STE20-type kinase TAOK3 regulates hepatic lipid partitioning
Source: Mol Metab. 2021 Oct 8;54:101353. doi: 10.1016/j.molmet.2021.101353 (PMC8567304; doi:10.1016/j.molmet.2021.101353)
Supplement: Multimedia component 1 [file mmc1.docx]

**Supplementary Table S1.** List of antibodies used for Western blot and immunofluorescence analysis

| **Type** | **Antibody name and catalogue number** | **Working dilution** | **Company** |  |
| --- | --- | --- | --- | --- |
| Primary | anti-TAOK3 (PA5-82574) | 1:500 | Invitrogen (Waltman, MA) | |
| antibody | anti-TAOK3 (PA5-48843) | 1:200 | Invitrogen | |
|  | anti-ADRP (ab181452) | 1:500 | Abcam (Cambridge, UK) | |
|  | Anti-actin (sc-8432) | 1:500 | Santa Cruz Biotechnology (Santa Cruz, CA) | |
|  | anti-8-oxoG (ab62623) | 1:500 | Abcam | |
|  | anti-4-HNE (ab46545) | 1:500 | Abcam | |
|  | anti-E06 (330001S) | 1:100 | Avanti Polar Lipids, Inc. (Alabaster, AL) | |
|  | anti-KDEL (ab176333) | 1:1000 | Abcam | |
|  | anti-CHOP (MA1-250) | 1:200 | Invitrogen | |
|  | anti-PEX5 (PA5-58716)  anti-PMP70 (PA1-650) | 1:500  1:500 | Invitrogen  Invitrogen | |
|  | anti-JNK (#9252) | 1:500 | Cell Signaling Technology (Boston, MA) | |
|  | anti-p-JNK (sc-293136) | 1:200 | Santa Cruz Biotechnology | |
|  | anti-YAP (#8418) | 1:1000 | Cell Signaling Technology | |
|  | anti-p-YAP (#4911) | 1:1000 | Cell Signaling Technology | |
|  | anti-STK25 (25821-1-AP)  anti-MST3 (#3723)  anti-MST4 (#3822)  anti-GAPDH (sc-47724) | 1:750  1:1000  1:1000  1:1000 | Proteintech (Chicago, IL)  Cell Signaling Technology  Cell Signaling Technology  Santa Cruz Biotechnology | |
|  | anti-MYC (#2276) | 1:1000 | Cell Signaling Technology | |
|  | anti-MYC (PA1-981) | 1:100 | Invitrogen | |
|  | anti-FLAG (PA1-984B) | 1:500 | Invitrogen | |
| Secondary antibody | Alexa Fluor-488-labeled anti-mouse IgG (A11029) | 1:500 | Invitrogen | |
|  | Alexa Fluor-488-labeled anti-rabbit IgG (A11008) | 1:500 | Invitrogen | |
|  | Alexa Fluor-594-labeled anti-mouse IgG (A11005) | 1:500 | Invitrogen | |
|  | Alexa Fluor-594-labeled anti-rabbit IgG (A21207)  anti-rabbit IgG (#7074) | 1:500  1:1000 | Invitrogen  Cell Signaling Technology | |
|  | anti-mouse IgG (#7076) | 1:1000 | Cell Signaling Technology | |
